# Association of Recreational Cannabis Legalization With Maternal Cannabis Use in the Preconception, Prenatal, and Postpartum Periods

**DOI:** 10.1001/jamanetworkopen.2021.0138

**Published:** 2021-02-25

**Authors:** Kara R. Skelton, Amelie A. Hecht, Sara E. Benjamin-Neelon

**Affiliations:** 1 Department of Health, Behavior and Society, Johns Hopkins Bloomberg School of Public Health, Baltimore, Maryland; 2 Department of Health Sciences, Towson University College of Health Professions, Towson, Maryland; 3 Department of Health Policy and Management, Johns Hopkins Bloomberg School of Public Health, Baltimore, Maryland

## Abstract

**Importance:**

Recent studies have revealed increases in population-level cannabis use after legalization of recreational cannabis. However, the association of cannabis legalization with maternal cannabis use during important life stages remains unknown.

**Objective:**

To investigate the association of legalization of recreational cannabis with maternal cannabis use during the preconception, prenatal, and postpartum periods.

**Design, Setting, and Participants:**

This repeated cross-sectional study used state-level data on women who delivered live-born infants in the US from the Pregnancy Risk Assessment Monitoring System from January 2004 to December 2018. Data from 2 states that had legalized recreational cannabis (Alaska and Maine) and 2 states that had not legalized recreational cannabis (New Hampshire and Vermont) were used. Women completed surveys 2 to 6 months after delivery, reporting preconception, prenatal, and postpartum cannabis use.

**Exposure:**

State recreational cannabis legalization.

**Main Outcomes and Measures:**

The 3 primary outcomes were self-reported cannabis use during the 12 months before pregnancy (preconception), during pregnancy (prenatal), and the 2 to 6 months after pregnancy (postpartum). A difference-in-differences analysis was used to compare changes in the prevalence of maternal cannabis use during each period before and after state legalization of recreational cannabis, controlling for maternal characteristics (age, race/ethnicity, educational level, income, cigarette smoking, and breastfeeding) and state fixed effects. State-specific survey weights were used.

**Results:**

The analytic sample included 23 082 women in the preconception period, 23 859 in the prenatal period, and 26 610 in the postpartum period. In each analysis, most women were married (range among all groups, 63.9%-64.8%), aged 25 to 34 years (preconception, 55.4%; prenatal, 55.9%; postpartum, 56.1%), and had an annual household income less than $50 000 (preconception, 55.7%; prenatal, 56.3%; postpartum, 55.5%). In adjusted analyses, preconception and postpartum cannabis use increased significantly in states that had legalized recreational cannabis compared with states that had not legalized it (preconception risk difference, 0.0457 [95% CI, 0.0013-0.0900]; *P* = .04; postpartum risk difference, 0.0539 [95% CI, 0.0259-0.0818]; *P* < .001). The risk difference for prenatal cannabis use was not significant (0.0070; 95% CI, −0.0120 to 0.0260; *P* = .47).

**Conclusions and Relevance:**

In this repeated cross-sectional study, recreational cannabis legalization was associated with changes in maternal cannabis use before and after pregnancy. The findings suggest that future studies should undertake an interdisciplinary approach to maximize benefit and application of findings to future public health, health care, and policy sectors.

## Key Points

**Question:**

Is there an association between recreational cannabis legalization and prevalence of maternal cannabis use during the preconception, prenatal, and postpartum periods?

**Findings:**

In this cross-sectional study of 73 551 women, cannabis use increased significantly among women before pregnancy and after pregnancy but not during pregnancy in states that had legalized recreational cannabis compared with states that had not legalized cannabis.

**Meaning:**

These findings suggest the need for interdisciplinary research to better understand how recreational cannabis policies are associated with maternal cannabis use.

## Teaser

This cross-sectional study examines the association between state legalization of recreational cannabis and prevalence of maternal cannabis use during the preconception, prenatal, and postpartum periods.

## Introduction

Levels of safe prenatal cannabis use have not been established to date,^1^ and questions regarding the risk of cannabis use have been the subject of debate.^2^ However, state-level recreational cannabis legalization in the US, particularly in states with policies that include provisions for cannabis commercialization, make cannabis products more attainable, affordable, and socially acceptable, which may be contributing to increased use.^3,4^

Cannabis use among women of reproductive age and pregnant women in the US has steadily increased over the past 2 decades.^5,6^ This increased use may be associated with an array factors at both individual and ecological levels, including decreased risk perceptions associated with cannabis use and increased social acceptance of use.^7,8,9^ In addition, recent evidence supports hypotheses that increases in cannabis use may be explained by recreational and not medicinal use.^10,11^

Under US federal law, cannabis possession and use remain illegal.^12^ However, the legality of cannabis use is rapidly changing at the state level. As of 2020, 15 states and the District of Columbia had legalized recreational cannabis for adults 21 years and older. Initially, recreational cannabis legalization was a result of voter support, which has doubled since the early 2000s.^3^ More recently, however, the political climate in the US has led to increased legislative support for expansive cannabis reform and legalization.^13,14^ This support is evidenced by legalization of recreational cannabis through state legislatures, current pending legislation, and numerous states in which legislative bodies have introduced bills in the past 2 years.^13,14^ Increases in legislative support, in particular, create an urgent need to examine the public health effects of recreational cannabis legalization, including the association of legalization with maternal and child health.

The extent to which changing recreational cannabis policies are associated with maternal cannabis use during critical maternal and child health periods is unclear. A recent cross-sectional study^15^ revealed that women residing in states in which recreational cannabis use is legal were significantly more likely to use cannabis during these critical periods compared with women residing in states yet to legalize recreational cannabis. Another recent case study^16^ in Colorado revealed that prenatal use increased after recreational use of cannabis had been legalized. Most existing evidence is restricted by study design (eg, cross-sectional) or narrow geographic focus (eg, specific state), limiting generalizability of findings.^16^ In a recent review^17^ of the prevalence of cannabis use among women during the prenatal period, the investigators found insufficient evidence to assess the association of recreational cannabis legalization with prenatal cannabis use. Cumulatively, prior literature supports the need for more robust evidence examining associations between recreational cannabis legalization and maternal cannabis use during important maternal and child health periods.^15,16,17^

To address this gap, we examined whether state recreational cannabis legalization was associated with changes in maternal cannabis use during the preconception, prenatal, and postpartum periods. This natural experiment was possible through geographic and temporal variations in state-level recreational cannabis legalization in the US. On the basis of prior research,^15,16^ we hypothesized that recreational cannabis legalization would be associated with increases in maternal cannabis use during each period of interest.

## Methods

### Overview

This repeated cross-sectional study used a difference-in-differences approach with data from the Pregnancy Risk Assessment Monitoring System (PRAMS) from January 2004 to December 2018. The Centers for Disease Control and Prevention (CDC) institutional review board approved this study; the Johns Hopkins Bloomberg School of Public Health institutional review board provided a study exemption because the study was considered non–human participant research and consisted of secondary analysis of deidentified data. For PRAMS, informed consent is not required because it is implied by completion of the survey by the respondent.^18^ This study followed the Strengthening the Reporting of Observational Studies in Epidemiology (STROBE) guideline.

### Data Source

The PRAMS is a state-specific, population-based surveillance system of the CDC, covering approximately 83% of all US births.^18^ Participating PRAMS states follow a standardized data collection protocol that uses birth certificates as the sampling frame to identify women delivering a live-born infant during the corresponding surveillance year.^18^ Eligible women are randomly sampled between 2 and 6 months after delivery by mail or telephone and asked about maternal behaviors and experiences before, during, and after pregnancy.^18^ The CDC releases data for PRAMS states that meet the response rate threshold, which has decreased from 70% in the early 2000s to 55% in the most recent phase (phase 8).^18^ The PRAMS methods, which detail variations to the standardized methods and procedures over time, have been published elsewhere.^18,19^

### Exposure

The primary exposure was state-level recreational cannabis legalization. We compared changes in self-reported cannabis use among women living in states where recreational cannabis was legal with that among women living in states that have not legalized recreational cannabis. Over the 15-year study period, a small number of PRAMS states included questions about cannabis use in their annual surveys. Among these states, we included only those that asked respondents about cannabis use during all 3 periods of interest and had data available before and after legalization of recreational cannabis. Alaska and Maine were the only 2 states meeting the inclusion criteria that had legalized recreational cannabis (intervention states). Colorado and Washington had postlegalization data only; Michigan had prelegalization data only. New Hampshire and Vermont were the only states with data available for similar prelegalization and postlegalization periods for comparison (comparison states).

Data availability in the periods before and after legalization differed across states because of variations in policy enactment dates, cannabis decriminalization enactment dates, and inclusion of cannabis questions in annual surveys. Alaska passed recreational cannabis legislation in November 2014; Maine passed recreational cannabis legislation in December 2016. Using legislation-effective dates, we created a state-level indicator variable that represented the individualized prelegalization and postlegalization periods for Alaska and Maine. Of note, Alaska and Maine decriminalized cannabis in 1975 and 2009, respectively—years before legalization. Because New Hampshire and Vermont had not legalized but had decriminalized cannabis, we used the same strategy to code these comparison states. Thus, for this analysis, we had approximately 9 years of data for comparison states (2009-2017) and 14 years of data for intervention states (2004-2018) (Table 1).

**Table 1.** Periods Before and After Legalization of Recreational Cannabis Use for States Included in the Study

State	Before legalization	After legalization
Alaska^a^	January 2004 to January 2015	March 2015 to December 2018
Maine^b^	January 2016 to December 2016	February 2017 to December 2018
New Hampshire^c^	January 2016 to December 2016	January 2017 to August 2017
Vermont^d^	January 2009 to December 2011	January 2012 to July 2013

^a^ Alaska passed recreational cannabis legalization on November 4, 2014, with an effective date of February 24, 2015.

^b^ Maine passed recreational cannabis legislation on December 17, 2016, with an effective date of January 30, 2017.

^c^ New Hampshire decriminalized cannabis (effective) in September 2017. The decriminalization date served as the demarcation date for this analysis.

^d^ Vermont decriminalized cannabis in August 2013. The decriminalization date served as the demarcation date for this analysis.

To create our study sample for each period, we assessed whether each period fell entirely in the prelegalization or postlegalization window using the infant’s date of birth and gestational age. We excluded women if the corresponding period crossed the legalization-effective date. For example, if a woman’s preconception period crossed the effective date but the prenatal and postpartum periods fell entirely after legalization, we excluded the preconception data from analysis but included the data from the prenatal and postpartum periods.

### Outcome

The 3 primary outcomes of interest for this study were (1) preconception cannabis use, (2) prenatal cannabis use, and (3) postpartum cannabis use. The PRAMS asked respondents, “During any of the following time periods, did you use marijuana or hash in any form?” The question had the following response options: during the 12 months before I got pregnant (preconception), during my most recent pregnancy (prenatal), and since my new baby was born (postpartum). We then created an individual-level binary variable for use or no use for each period. When cannabis use was reported, recreational cannabis legalization had been in effect for at least 2 months.

### Covariates

We adjusted for individual-level covariates selected a priori that have been found to be associated with maternal cannabis use.^15,20,21,22^ These included maternal age; race/ethnicity; educational level; marital status; household income; participation in the Special Supplemental Nutrition Program for Women, Infants, and Children; and cigarette smoking during the corresponding period. Response options for household income on the annual PRAMS survey changed over time and across states. In cases in which the response options did not align with the cutoff of $50 000, we used the point closest to $50 000 (eg, for phase 7, we used<$52 000 for contiguous states). Because there was a low percentage (range, 0.01%-7.76%) of missingness among covariates, we imputed the weighted mode value separately for missing covariates for intervention and comparison states. Sensitivity analyses comparing results from complete cases vs imputed data showed that imputation did not meaningfully alter the direction or magnitude of findings.

### Statistical Analysis

We used a difference-in-differences approach^23^ to compare changes over time in maternal cannabis use in women in intervention states vs those in comparison states separately for the preconception, pregnancy, and postpartum periods. We used indicators for period, legalization status, and interaction between time and legalization status (difference-in-difference indicator) and controlled for state-level fixed effects in each analysis. To compare differences in risk of cannabis use for women in intervention and comparison states, we estimated unadjusted and adjusted risk differences and risk ratios separately for each period using a statistical significance threshold of *P* < .05 for 2-sided tests. We used state-specific survey weights to account for the complex sampling design of PRAMS, including nonresponse and noncoverage.^18^ Therefore, we considered that variations in annual response rates for states were accounted for in this weighting process. We used Stata, version 16.1 (StataCorp Inc) for all analyses.

## Results

Our final analytic sample women who delivered live-born infants included 23 082 participants in the preconception period, 23 859 in the prenatal period, and 26 610 in the postpartum period. In each analysis, most women were married (range among all groups, 63.9%-64.8%), were aged 25 to 34 years (preconception, 55.4%; prenatal, 55.9%; postpartum, 56.1%), and had an annual household income less than $50 000 (preconception, 55.7%; prenatal, 56.3%; postpartum, 55.5%) (Table 2). We found substantial imbalance among covariates between women in intervention and comparison states in each of the 3 analyses (Table 2).

**Table 2.** Characteristics of Women in the Preconception, Prenatal, and Postpartum Analysis by State Cannabis Law Status

Characteristic	Women, No. (%)					
	Preconception period (n = 23 082)^a^		Prenatal period (n = 23 859)^a^		Postpartum period (n = 26 610)^a^	
	Comparison (n = 5466)	Intervention (n = 17 616)	Comparison (n = 5642)	Intervention (n = 18 217)	Comparison (n = 6830)	Intervention (n = 19 780)
State^b^						
Alaska	0	15 960 (85.4)	0	16 335 (84.2)	0	17 274 (80.9)
Maine	0	1656 (14.7)	0	1882 (15.8)	0	2506 (19.2)
New Hampshire	975 (45.0)	0	576 (70.5)	0	955 (37.2)	0
Vermont	4491 (55.0)	0	5066 (29.5)	0	5875 (62.8)	0
Married	3495 (64.8)	10 513 (64.1)	3605 (63.9)	10 906 (64.1)	4359 (64.4)	11 816 (63.9)
Age, y^b^						
≤17	62 (1.1)	425 (2.2)	64 (1.1)	416 (2.1)	73 (1.1)	441 (2.0)
18-24	1296 (21.6)	5562 (30.4)	1331 (22.1)	5655 (29.7)	1604 (22.0)	6081 (29.5)
25-34	3121 (59.5)	9187 (54.2)	3234 (59.5)	9591 (55.1)	3927 (50.3)	10 461 (55.1)
≥35	987 (17.8)	2439 (13.2)	1013 (17.3)	2552 (13.2)	1226 (17.7)	2794 (13.4)
Income ≥$50 000^b^^,^^c^	2425 (51.7)	6120 (42.1)	2435 (49.0)	6391 (42.3)	3006 (50.3)	7031 (42.8)
Non-Hispanic White^b^	5047 (91.3)	7925 (61.4)	5226 (92.2)	8288 (62.0)	6314 (91.5)	9190 (62.9)
At least some college^b^	3698 (69.5)	8476 (53.2)	3748 (67.3)	8799 (53.3)	4607 (68.5)	9749 (52.2)
WIC participation^d^	2686 (61.3)	9444 (52.7)	2526 (54.0)	9892 (54.0)	3161 (57.5)	10 786 (54.3)
Cigarette smoking^b^	1575 (25.3)	5440 (27.6)	961 (15.2)	3099 (14.0)	1351 (17.2)	4241 (18.5)

Abbreviation: WIC, Special Supplemental Nutrition Program for Women, Infants, and Children.

^a^ Number reflects unweighted raw counts, and percentage reflects survey-weighted proportions.

^b^ Statistically significant difference between intervention and comparison samples during all periods.

^c^ Income categories varied across states and survey years—phases 5, 6, and 8 (Alaska only): less than $50 000 and greater than or equal to $50 000; phase 7: less than $52 000 and greater than or equal to $52 001 for contiguous states and less than $56 000 and greater than or equal to $56 001 for Alaska; phase 8 (contiguous states): less than $48 000 and greater than or equal to $48 001.

^d^ Statistically significant difference between intervention and comparison samples during preconception and postpartum periods only.

Table 3 presents the primary findings for this analysis, including unadjusted and adjusted risk differences and risk ratios comparing self-reported cannabis use in states with and without recreational cannabis legalization. In unadjusted analyses, cannabis use increased significantly in intervention states compared with states that had not legalized recreational cannabis in the postpartum period only (risk difference, 0.0497; 95% CI, 0.0217-0.0776; *P* < .001). In adjusted analyses for the preconception and postpartum periods, cannabis use increased significantly in intervention states compared with states that had not legalized recreational cannabis (preconception risk difference, 0.0457 [95% CI, 0.0013-0.0900]; *P* = .04; postpartum risk difference, 0.0539 [95% CI, 0.0259-0.0818]; *P* < .001). The risk ratio in preconception and postpartum periods was also significant in adjusted analyses (preconception risk ratio, 1.2789 [95% CI, 1.0286-1.5901]; *P* = .03; postpartum risk ratio, 1.8316 [95% CI, 1.3962-2.4027]; *P* < .001). In adjusted analyses, the risk difference and risk ratio in the prenatal period were not significant (risk difference, 0.0070 [95% CI, −0.0120 to 0.0260]; *P* = .47; risk ratio, 1.11054 [95% CI, 0.8467 to 1.4432]; *P* = .46).

**Table 3.** Unadjusted and Adjusted Risk Differences and Risk Ratios of Cannabis Use During the Preconception, Prenatal, and Postpartum Periods by Recreational Cannabis Legalization Status

Cannabis use	Unadjusted				Adjusted^a^	
	Predicted probability		Difference from baseline (95% CI)	*P* value	Difference from baseline (95% CI)	*P* value
	Baseline (95% CI)	Follow-up (95% CI)				
**Preconception period**						
States						
Intervention	0.1576 (0.1499 to 0.1652)	0.2073 (0.1871 to 0.2276)	0.0498 (0.0285 to 0.0710)	<.001^b^	0.0773 (0.0560 to 0.0985)	<.001^b^
Comparison	0.1829 (0.1706 to 0.1952)	0.2061 (0.1679 to 0.2443)	0.0232 (−0.0167 to 0.0631)	.26	0.0279 (−0.0097 to 0.0655)	.15
Risk difference	NA	NA	0.03 0.0275 (−0.0155 to 0.0705)	.21	0.0457 (0.0013 to 0.0900)	.04^b^
Risk ratio	NA	NA	1.1661 (0.9293 to 1.4632)	.18	1.2789 (1.0286 to 1.5901)	.03^b^
**Prenatal period**						
States						
Intervention	0.0622 (0.0566 to 0.0678)	0.0865 (0.0763 to 0.0966)	0.0243 (0.0124 to 0.0361)	<.001^b^	0.0365 (0.0238 to 0.0493)	<.001^b^
Comparison	0.0588 (0.0502 to 0.0674)	0.0782 (0.0643 to 0.0920)	0.0194 (0.0033 to 0.0354)	.02^b^	0.0229 (0.0083 to 0.0375)	.002^b^
Risk difference	NA	NA	0.0032 (−0.0158 to 0.0221)	.74	0.0070 (−0.0120 to 0.0260)	.47
Risk ratio	NA	NA	1.0474 (0.7978 to 1.3750)	.74	1.1054 (0.8467 to 1.4432)	.46
**Postpartum period**						
States						
Intervention	0.0669 (0.0611 to 0.0727)	0.1050 (0.0951 to 0.1149)	0.0381 (0.0266 to 0.0496)	<.001^b^	0.0506 (0.0384 to 0.0629)	<.001^b^
Comparison	0.0747 (0.0640 to 0.0856)	0.0664 (0.0545 to 0.0783)	−0.0082 (−0.0260 to 0.0095)	.36	−0.0024 (−0.0188 to 0.0141)	.78
Risk difference	NA	NA	0.0497 (0.0217 to 0.0776)	<.001^b^	0.0539 (0.0259 to 0.0818)	<.001^b^
Risk ratio	NA	NA	1.7579 (1.3325 to 2.3190)	<.001^b^	1.8316 (1.3962 to 2.4027)	<.001^b^

Abbreviations: NA, not applicable; WIC, Special Supplemental Nutrition Program for Women, Infants, and Children.

^a^ Adjusted for race/ethnicity (non-Hispanic White or race/ethnicity other than non-Hispanic and White), marital status (married or not married), educational level (some college or more), income (≥$50 000), WIC participation (yes or no), maternal age (≤17 years, 18-24 years, 25-34 years, or ≥35 years), cigarette smoking during the associated period, and state fixed effects. Postestimation commands were used to calculate adjusted risk ratio and risk differences and linearized standard errors. Survey weights were used to account for the complex sampling design of the Pregnancy Risk Assessment Monitoring System, including nonresponse, sampling weight, and noncoverage.

^b^ Indicates statistical significance at *P* < .05.

## Discussion

In this repeated cross-sectional study, we examined the association between state recreational cannabis legalization and maternal cannabis use during the preconception, prenatal, and postpartum periods. Using data from PRAMS spanning approximately 15 years, we found that preconception and postpartum maternal cannabis use significantly increased in states that legalized recreational cannabis compared with states that had had not legalized it. Contrary to our hypotheses, prenatal cannabis use was not increased among women in intervention states compared with women in states that had not legalized recreational cannabis. Because emerging data suggest that adverse maternal and child health outcomes are associated with cannabis use,^16,17,20,24,25,26,27^ these study findings support maternal cannabis use as an important public health concern.

Our results build on previous research,^15,16,28^ providing support for an association between recreational cannabis legalization and perinatal cannabis use. However, most prior studies of cannabis use have focused on a single state,^16,29^ single period,^16^ or single year.^15^ This study was, to our knowledge, the first to assess associations between recreational cannabis legalization and maternal cannabis use during multiple maternal health periods in more than 1 state.

The risk difference for prenatal cannabis use was not significant, and several explanations are possible. First, we observed significant increases in self-reported prenatal cannabis use in intervention and comparison groups, reflecting a national trend of increased prenatal cannabis use over the past 2 decades.^5,10,30^ A second explanation is that underreporting of cannabis use in this period contributed to the null findings. In recent years, increasing public health concern about prenatal cannabis use has garnered national attention from leading maternal and child health organizations and the US Surgeon General.^1,31,32^ In turn, this national attention could have dissuaded women across the entire sample from disclosing prenatal cannabis use. States in which recreational cannabis use has been legalized typically use a portion of revenue generated from cannabis sales for public health prevention campaigns, with many states focusing on prenatal cannabis use when creating these large-scale health communication campaigns.^33,34^ However, women residing in states where recreational cannabis is illegal have poor or uncertain knowledge of the risks associated with cannabis use during pregnancy.^35,36^ State prenatal urine drug screening requirements have changed as a result of recreational cannabis legalization.^37^ Fear of punitive action—particularly among those who have had a positive toxicology screening result during pregnancy or at the time of delivery—may have caused women to be less likely to report use when asked retrospectively. Therefore, we hypothesized that women in states where recreational cannabis is legal may have been more aware of risks of prenatal cannabis use (eg, possible involvement of child protective services, adverse health effects) than women in states that had not legalized recreational use, resulting in substantial underreporting of prenatal cannabis use. Additional research to investigate this hypothesis is warranted, especially for future studies that include self-reported data.

Consensus among health care and public health professionals has not been reached regarding the health risks associated with perinatal cannabis use, which may be associated with an array of factors.^26,38,39,40^ First, there have been increases in the mean potency of Δ-9-tetrahydrocannabinol of nearly 3-fold over recent decades,^41^ which makes the results of some earlier studies less applicable. Second, most states that have legalized recreational cannabis also include provisions for cannabis commercialization, resulting in both new modes of administration and novel, more potent cannabis products.^33,42,43^ Third, issues with study methods, including uncontrolled confounding, also contribute to uncertainty regarding the evidence.^21^ However, findings from recent studies examining the association of cannabis use with female reproductive system health, perinatal outcomes, and infant and child neurodevelopment have elicited concern.^16,17,20,24,25,26^

Chemical substances in cannabis (Δ-9-tetrahydrocannabinol, cannabidiol) stimulate the endocannabinoid system,^44^ which is vital in homeostasis and maintenance of organ systems, including the reproductive system.^44^ Thus, preconception or prenatal exposure to cannabis may lead to disruptions in ovulatory processes, implantation, and fetal and infant neurologic development.^45,46^ Evidence from studies conducted over the past decade supports increased risk of adverse perinatal outcomes associated with prenatal cannabis use.^16,20,26,28,47,48,49,50^ In several studies,^39,47,51^ investigators found an association between prenatal cannabis use and admission to the neonatal intensive care unit. National pediatric and women’s health organizations are united in their concern regarding cannabis use during pregnancy and lactation, particularly in relation to fetal growth and neurodevelopment, and thus recommend avoidance of cannabis use during these critical periods.^1,32,38,52^

Given our finding that there was a higher prevalence of preconception and postpartum cannabis use in states in which recreational cannabis has been legalized, adverse reproductive health outcomes may be more frequent in these states. Adjusted analyses revealed that women who resided in intervention states were 1.2789 times more likely to use cannabis during the preconception period and 1.8316 times more likely to use cannabis during the postpartum period compared with women in states where cannabis was not legal. The use of other substances, such as tobacco or opioids,^9,53,54^ in addition to cannabis suggests that increases in maternal cannabis use associated with recreational cannabis legalization may also be associated with a concurrent increase in use of other substances.^55^ Given the changing legislative landscape in relation to cannabis in the US, our findings, along with evidence that preconception cannabis use is associated with prenatal cannabis use,^30^ suggest the need for tailored cannabis use prevention interventions targeted to all women of reproductive age.

Results from this study provide the framework from which future research can potentially build. Many specific policies under the umbrella of recreational cannabis legalization may have an effect on maternal cannabis use. Thus, future research should assess whether policies such as commercialization, on-site consumption, or delivery affect patterns of maternal cannabis use. This research could reveal policies associated with reduction of cannabis use during these critical periods. Furthermore, understanding how maternal cannabis use changes during the intermediary period of cannabis legalization (between law passage and effective date) warrants future study. Ultimately, the novelty of recreational cannabis policies creates a need for additional high-quality research in this area, for which we recommend an interdisciplinary approach to maximize benefit and application of findings to the public health, health care, and policy sectors.

### Limitations

This study has limitations. PRAMS data availability and the number of states that included survey questions about cannabis use reduced the number of states eligible for inclusion and also resulted in inadequate follow-up periods, particularly for the postpartum analysis. Intervention and comparison states were not perfectly matched, which was expected because of differences in PRAMS sampling strategies across states. These issues limit the generalizability of results to states included in the analysis. Because cannabis use is likely associated with likelihood of abortions or miscarriages,^53^ generalizability of results is limited to women who had live births only as opposed to all women of reproductive age. Prevalence estimates of cannabis use in this analysis are likely conservative. Our outcome of interest was self-reported, which underestimates cannabis use compared with estimates obtained using biochemical measures.^6,25^ Social desirability and retrospective recall bias (women are asked about cannabis use 2-6 months after they give birth) likely affected disclosure of cannabis use. For survey years during which cannabis was illegal, as well as for women younger than 21 years (for whom recreational cannabis remains illegal in all states), fear of punitive action likely contributed to underreporting. It is also plausible that women in states that legalized recreational cannabis felt more comfortable disclosing use after policy enactment, leading to the conclusion that our findings were not a result of a change in cannabis use prevalence but instead the result of a greater willingness to report use. We were unable to differentiate women who used cannabis for an approved medical condition from those who used it for recreational purposes. However, recent nationally based estimates have revealed medicinal cannabis use among pregnant women and nonpregnant women to be low— 0.7% and 1.1%, respectively.^10^ We acknowledge that states in which cannabis has been legalized may be systematically different from those that have not legalized it. However, Vermont legalized recreational cannabis in January 2018, and New Hampshire had legislation pending in the Senate recently,^14^ revealing similar legislative and public attitudes regarding recreational cannabis in comparison states. The inclusion of Alaska could be problematic given geographic and other differences in states included for comparison.

## Conclusions

In this repeated cross-sectional study, preconception and postpartum cannabis use, but not prenatal cannabis use, increased significantly in states that had legalized recreational cannabis compared with states that had not legalized cannabis. Results of this study suggest the need for high-quality studies that aim to examine the health effects associated with cannabis use during these periods. Future research should also prospectively examine how specific cannabis policies (eg, legalization, commercialization, and on-site consumption) affect maternal use patterns. These studies are needed to ensure that voters and policy makers are fully informed when making decisions regarding cannabis legality so that maternal and child health is safeguarded.
